# “Bow-tie” optimal pathway discovery analysis of sepsis hospital admissions using the Hospital Episode Statistics database in England

**DOI:** 10.1093/jamiaopen/ooaa039

**Published:** 2020-09-20

**Authors:** Hugo De Oliveira, Martin Prodel, Ludovic Lamarsalle, Matt Inada-Kim, Kenny Ajayi, Julia Wilkins, Sara Sekelj, Sue Beecroft, Sally Snow, Ruth Slater, Andi Orlowski

**Affiliations:** o1 HEVA, Lyon Cedex 06, France; o2 Mines Saint-Etienne, Univ Clermont Auvergne, CNRS, UMR 6158 LIMOS, Centre CIS, Saint-Etienne, France; o3 Hampshire Hospitals NHS Foundation Trust, Royal Hampshire County Hospital, Winchester, Hampshire, UK; o4 NHS England & Improvement, London, UK; o5 Wessex Academic Health & Science Network, Innovation Centre, Chilworth, Hampshire, UK; o6 Imperial College Health Partners, London, UK; o7 Harvey Walsh Limited, C/O Legalinx Limited, Cardiff, UK; o8 Department of Primary Care and Public Health, Imperial College London, London, UK

**Keywords:** data mining, process mining, hospitals/statistics and numerical data, HES database, sepsis

## Abstract

**Objective:**

The “Bow-tie” optimal pathway discovery analysis uses large clinical event datasets to map clinical pathways and to visualize risks (improvement opportunities) before, and outcomes after, a specific clinical event. This proof-of-concept study assesses the use of NHS Hospital Episode Statistics (HES) in England as a potential clinical event dataset for this pathway discovery analysis approach.

**Materials and Methods:**

A metaheuristic optimization algorithm was used to perform the “bow-tie” analysis on HES event log data for sepsis (ICD-10 A40/A41) in 2016. Analysis of hospital episodes across inpatient and outpatient departments was performed for the period 730 days before and 365 days after the index sepsis hospitalization event.

**Results:**

HES data captured a sepsis event for 76 523 individuals (>13 years), relating to 580 000 coded events (across 220 sepsis and non-sepsis event classes). The “bow-tie” analysis identified several diagnoses that most frequently preceded hospitalization for sepsis, in line with the expectation that sepsis most frequently occurs in vulnerable populations. A diagnosis of pneumonia (5 290 patients) and urinary tract infections (UTIs; 2 057 patients) most often preceded the sepsis event, with recurrent UTIs acting as a potential indicative risk factor for sepsis.

**Discussion:**

This proof-of-concept study demonstrates that a “bow-tie” pathway discovery analysis of the HES database can be undertaken and provides clinical insights that, with further study, could help improve the identification and management of sepsis. The algorithm can now be more widely applied to HES data to undertake targeted clinical pathway analysis across multiple healthcare conditions.

**LAY SUMMARY**

This “proof of concept” study assessed whether a new process mining algorithm can be successfully used with the Hospital Episode Statistics (HES) database in England. The HES database contains information on inpatient and outpatient hospital stays. The algorithm was applied to map out the most common medical diagnoses that a patient is likely to experience in advance of sepsis, and which diagnostic events most frequently occur afterwards. The sepsis diagnosis is a central “index” event and the algorithm is referred to as the “bow-tie” optimal pathway discovery analysis. The study period was 730 days before sepsis and 365 days afterwards and included anonymized data for individuals >13 years of age. Overall, 76 523 patient records were examined and visualized using the “bow-tie” methodology. The findings were consistent with what might be expected for patients experiencing sepsis and confirmed that the algorithm can be successfully used with the HES database. It provided a number of insights, for example the most common diagnoses before sepsis occurs, that could be explored further to help improve the clinical care of patients at high risk of sepsis. The algorithm can also now be used to explore other areas of clinical interest using the HES database.

## INTRODUCTION

### Process mining using pathway discovery analysis in healthcare

Process mining (PM) in a healthcare setting is gaining increasing focus due to the vast amounts of clinical data collected and stored in healthcare databases around the world.^1^^,^^2^ PM analyses can be used to map and study clinical pathways; an automated discovery process enables a descriptive “process model” to be extracted (discovered) using an “event log” taken from a specific healthcare database.^3^^,^^4^ The discovered model has the potential to improve our understanding of the steps involved in disease progression and the real-life behavior of specific patterns of care.^1–6^

The complex nature of many healthcare processes means that the use of PM methods with healthcare datasets can be challenging.^1^^,^^2^ Healthcare data are collected and stored at varying standards and complexity, and identifying which databases are best suited to analysis using PM methodologies remains under investigation. Equally, identifying the best PM methodologies for effectively extracting, “discovering” and visualizing the most relevant event data from such large and diverse healthcare datasets requires increasingly sophisticated algorithms and approaches.^7^

Many automated PM process discovery algorithms have been applied to a healthcare setting, including heuristic miner, fuzzy miner, and genetic miner applications.^1^^,^^2^^,^^8^ Many of these algorithms have been hampered by a range of limitations. The search-based genetic miner approach, for example,^8^ is limited by its scalability and practical ability to deal with the large datasets commonly seen in healthcare—where patient numbers and clinical events may number hundreds of thousands. Prodel et al^5^ have developed a proprietary search-based pathway discovery analysis procedure based on a metaheuristic optimization algorithm designed for healthcare that is scalable to population size. This metaheuristic approach uses a combination of Monte Carlo sampling and tabu search to overcome the complexity related to large event logs and uses a “replayability score” to determine the “fitness” of the discovered process model under specific size constraints (event nodes and edges included in the process model). It is particularly robust at dealing with the high levels of “noise” and complexity in the type of event logs extracted from healthcare databases. The Prodel et al algorithm can be used to provide a visual overview of risks (opportunities to improve) before a specific clinical event (the “index” event), such as a stroke, and outcomes after the event. Due to the way in which the pathways are visually represented this is introduced here, for the first time, as a “bow-tie” pathway discovery analysis. The Prodel et al algorithm has been implemented on the French PMSI hospital database to discover patterns in patient pathways for cardiovascular disease before and after implantation of a cardioverter defibrillator.^5^ As well as confirming existing cardiologist knowledge around the main steps in the patient pathway, the analysis also discovered additional outcomes related to cardiomyopathy, tachycardia, and ischemic disease.

### The potential for process mining the Hospital Episode Statistics database using a sepsis case study

Following initial validation with the French PMSI database, the next step is to assess the transferability of the Prodel et al algorithm to other healthcare databases. A key candidate is the Hospital Episode Statistics (HES) database—a data warehouse containing details of inpatient and outpatient appointments at NHS hospitals in England.^9^ The requirements of a PM event log are that: (1) each event refers to a unique activity (or step in the process e.g. a hospital stay); (2) each event can be linked to a consistent “trace” (eg, the patient ID); and (3) events have a time stamp allowing them to be sequentially ordered.^5^ HES data comply with each of these requirements, making it a suitable data source for PM analyses; however, to the best of our knowledge, no PM studies using HES data have been reported to date.

Sepsis is defined as “a life-threatening organ dysfunction caused by a dysregulated host response to an infection”.^10^ Sepsis occurs when the body’s immune response to an infection is out of balance, triggering a reaction that can damage multiple organ systems. If sepsis progresses to septic shock, this can lead to death. Many people who survive initial hospitalization for sepsis go on to experience high rates of healthcare utilization and medical complications, with relapse (recurrence or reinfection) rates of up to 60% in some settings.^11^^,^^12^ Sepsis is considered a major health burden and is thought to have been responsible for 11 million deaths worldwide during 2017 (equating to 20% of all global deaths).^13^ However, there is a lack of suitable metrics to identify sepsis (it is frequently confirmed retrospectively), and correct identification of patient risk factors and the ability to gain a timely diagnosis of “suspicion of sepsis” (SOS) remain significant challenges.^14^^,^^15^ PM analyses offer the opportunity to better understand sequences of events affecting patients in the time prior to sepsis hospitalization, and factors associated with outcomes after the event, including recurrence or reinfection. PM of a single hospital’s event log for patients entering the emergency room with sepsis was recently undertaken using a heuristic discovery algorithm in a small dataset (*n* = 1 050) from a Dutch hospital.^16^ As a key “index” event, sepsis specifically offers a valid case model for the Prodel et al pathway discovery algorithm approach.

### Objectives of this study

The current study used the Prodel et al^5^ metaheuristic optimization algorithm to perform a “bow-tie” pathway discovery analysis on event log data extracted from the HES database in order to:

1. Evaluate the application of a PM algorithm on a HES dataset, including technical aspects of data management
2. Perform a proof of concept pathway discovery case example on the index event of sepsis

## MATERIALS AND METHODS

### Data collection

An anonymized cohort was derived from the HES database for all patients in England with at least one hospital episode for sepsis (ICD-10 A40 or A41, and their derivatives) present in any diagnosis within an episode spell between January 1, and December 31, 2016. Data for patients aged >13 years of age at the time of their first sepsis episode were included. A deliberate decision to exclude the pediatric sepsis pathway was made in consultation with clinical specialists due to the high levels of critical care required for pediatric sepsis, and 13 years of age was felt to be the most appropriate cut off point.

Analysis of hospital episodes across inpatient and outpatient departments was performed for the 730 days before and 365 days after an individual’s first hospitalization for sepsis in 2016 (the index hospitalization). Hospital episode data were extracted regardless of the medical reason (even if unrelated to sepsis) to create the HES “unprocessed event log”. Hospital episodes are linked at record level by the individual’s pseudonymized HES ID number (the event log “trace”). A strict statistical disclosure control was applied in accordance with NHS Digital protocol. This suppresses small numbers to stop identification of individuals and ensures that patient confidentiality is maintained.

Mortality data (eg, death certificate data) are an important data point for confirmation of sepsis. However, HES data on death were excluded from the analysis because: (1) death can occur at any time (making it hard to isolate) and could not be specifically attributed to sepsis using HES data codes; and (2) not all deaths would occur (and be documented) in hospital, making this an incomplete dataset.

### Data structuration

As this was the first time the algorithm had been applied to HES data, a protocol was developed to structure the dataset, appropriately label inputs (hospital episodes/hospital stays), handle missing data, and classify and group events to create the final “coded event” log using ICD-10 codes (Table 1). In each hospital spell the primary diagnosis (main reason for the hospital stay) was used as the allocated diagnostic event (and other diagnosis made with the same hospital stay, or time stamp, were discarded).

**Table 1.** Data structuration protocol

Requirement	Structuration procedure
Handling missing values in the unprocessed HES dataset	Hospital stays with no discharge date and no length of stay (LOS) were labeled as day care (LOS < 1 day). 34 episodes not related to any hospital stay were removed from the dataset.
Removing redundant variables	Non-informative administrative or accounting variables captured in the unprocessed HES dataset were removed, as was demographic information.
Standardizing data input fields	Missing fields^a^ were left blank to enable all outpatient and inpatient episodes to be captured in one database.
Timestamp conversion	Timestamps were formatted into yyyy-mm-dd. The smallest unit considered was days.
Labeling and grouping diagnostic codes	Unprocessed medical data (HES episodes) were labeled using ICD-10 codes. Published methods were used to combine ICD-10 codes into one of 220 coded event classes.^17^^,^^18^
Creating a time-ordered event sequence for each HES ID	Episodes relating to a single stay were grouped into one coded event record (from the 220 ICD-10 coded events). A set of coded event records relating to each HES ID was time-ordered in a dataset.

a For example, LOS, discharge date and arrival method are included within HES for inpatient stays, but are not relevant for outpatient episodes.

To improve data manageability, the unprocessed HES diagnostic events were consolidated under 220 diagnostic “coded event” classes that represent the medical reason for the hospital stay. This followed the approach taken by Choi et al,^17^ who created grouped meta-codes (coded events), to improve the interpretability of large clinical datasets. The clinical classification software (CCS) for ICD-10-PCS was used for the meta-code grouping.^18^ Patient demographic information contained in the HES database, such as gender, age, or geographical information, was not analyzed. The data structuration protocol is now reproducible for additional HES datasets and can be fully automated in the future.

### Pathway discovery analysis using the metaheuristic algorithm

**Figure 1.**
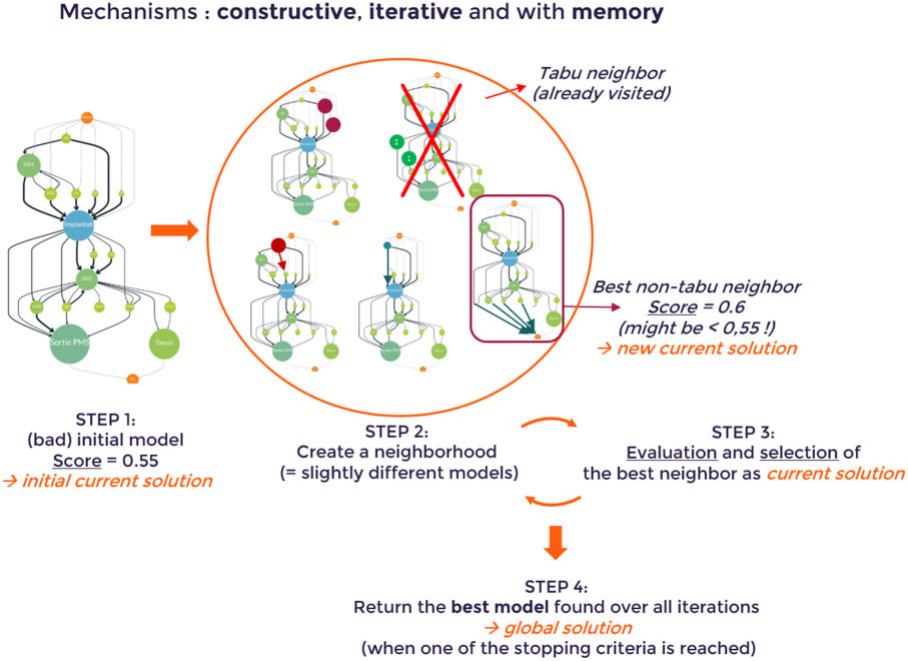
Constructive, iterative, optimal pathway discovery algorithm. The score for each model describes the representativeness of the model. A “new” current solution is only adopted if it scores more highly than the current model. All Tabu neighbors (those that have recently been visited) are censored.

The metaheuristic algorithm was used to create a “pathway” or process discovery model that best described the sequence of clinical events prior to and following the index hospitalization for sepsis (Figure 1).

With PM there is a trade-off between data presentation and interpretation for large, complex event logs such as healthcare datasets. To reduce complexity, candidate models were constrained to include no more than 25 coded event class “nodes” in the period prior to sepsis with up to 40 “links” connecting those nodes, and no more than 15 coded event class nodes with up to 25 connecting links after the index sepsis event (the complexity threshold). Each link (or edge) represents a time-ordered sequence of one coded event class node following another. The constraints on the size of the model is a guarantee to avoid overfitting, while limiting underfitting by ensuring fitness using a replayability score (see below).

During the initialization phase (Step 1), a starting model is chosen at random and given a replayability score to describe how well it represents the input dataset (from 0 to 1, where 1 is the highest possible score). This starting model becomes the current solution and the accepted “global solution” at Step 1. In subsequent iterations, small “smart” changes are made to the current solution to create sets of “neighbor” models (Step 2). Each iteration is able to reselect data from the full set of 220 diagnostic event codes. At each iteration, the best neighbor model (achieving the best replayability score) is selected as the new current solution (and censored from subsequent iterations). If this current solution is better (scores more highly) than the current global solution, then the old global solution is discarded and this new model takes its place (Step 3). Steps 2 and 3 are then repeated until one of the stopping criteria is reached: either (1) 25 iterations without improving the global solution or (2) reaching a total of 500 iterations. The global solution at the point of stopping is returned as the final “Sepsis Discovery Model”.

The Prodel et al metaheuristic algorithm, including data pre-processing and model construction, was implemented in Python 3.7 (further details are provided in Prodel et al, 2018).^5^

Visualization of the resulting model is achieved via a tablet application developed by the company HEVA for that purpose. The tablet application also enables quantification of patient numbers and visualization of patient flow from one diagnostic code to another.

## RESULTS

A total of 76 523 individuals aged >13 years at the time of their index sepsis event had a sepsis hospital episode in England in 2016. The time-ordered series of hospital episodes (an episode of care provided to a patient from admission to discharge) and hospital spells (the continuous stay of a patient in hospital using a hospital bed) were linked to individual HES IDs (patient traces), resulting in 580 000 coded events after data structuration and event labeling (Table 2).

**Table 2.** Hospital episode data included in the “bow-tie” analysis

Patients	Hospital episodes	Hospital spells	Coded events
76 523	4 509 000	964 000	580 000

Episodes, hospital spells, and coded events are the total combined number of events from the analyzed period: 2 years prior and 1 year after the index hospitalization for sepsis.

**Figure 2.**
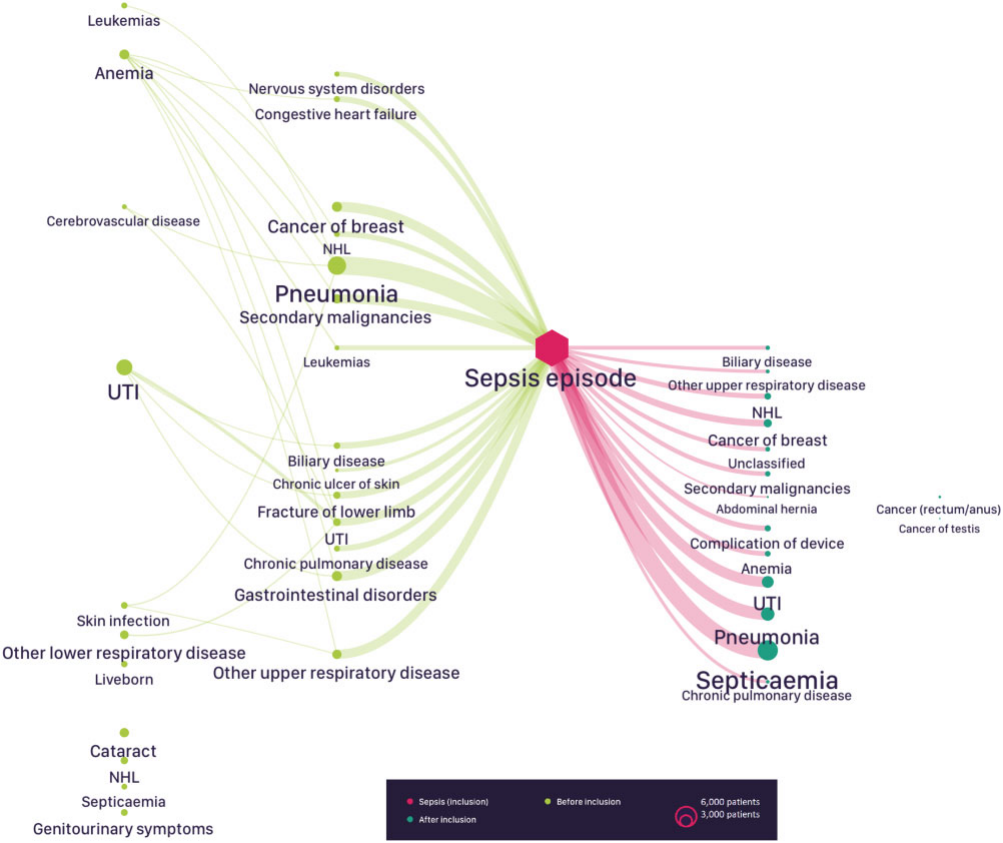
Bow-tie graph of the coded events in the 2 years before and 1 year after the index hospitalization for sepsis. The “bow-tie” graph is read from left to right, with circles representing event nodes of the process model (ie, coded events). The links (or edges) from each circle represent the time-ordered sequence of one coded event node following another. The sizes of nodes and links are proportional to the number of patients following this pathway. *Note*: The coded event “septicemia” contains a number of additional sepsis-related codes in addition to A40 or A41 (and their derivatives). See Supplementary Materials for full details of the HES ICD-10 codes included in this coded event.

Figure 2 shows the “bow-tie” graph of the most prominent coded events in the “final sepsis discovery model” for the patient cohort in the 2 years before and 1 year after the index hospitalization for sepsis.

A diagnosis of cancer, particularly non-Hodgkin’s lymphoma (1 409 patients), cancer of the breast (2 753 patients), leukemia (1 520 patients), and secondary malignancies (2 492 patients), or of gastrointestinal disorders (2 742 patients), pneumonia (5 290 patients), or urinary tract infections (UTIs; 2 057) most often directly preceded the index hospitalization for sepsis (Figure 3A). It should be noted that the analysis can only assess that a stay related to the cancer diagnosis occurred in advance of the index sepsis event, and does not provide any precision as to the reason (the cancer itself or the treatment) leading to the sepsis admission.

**Figure 3.**
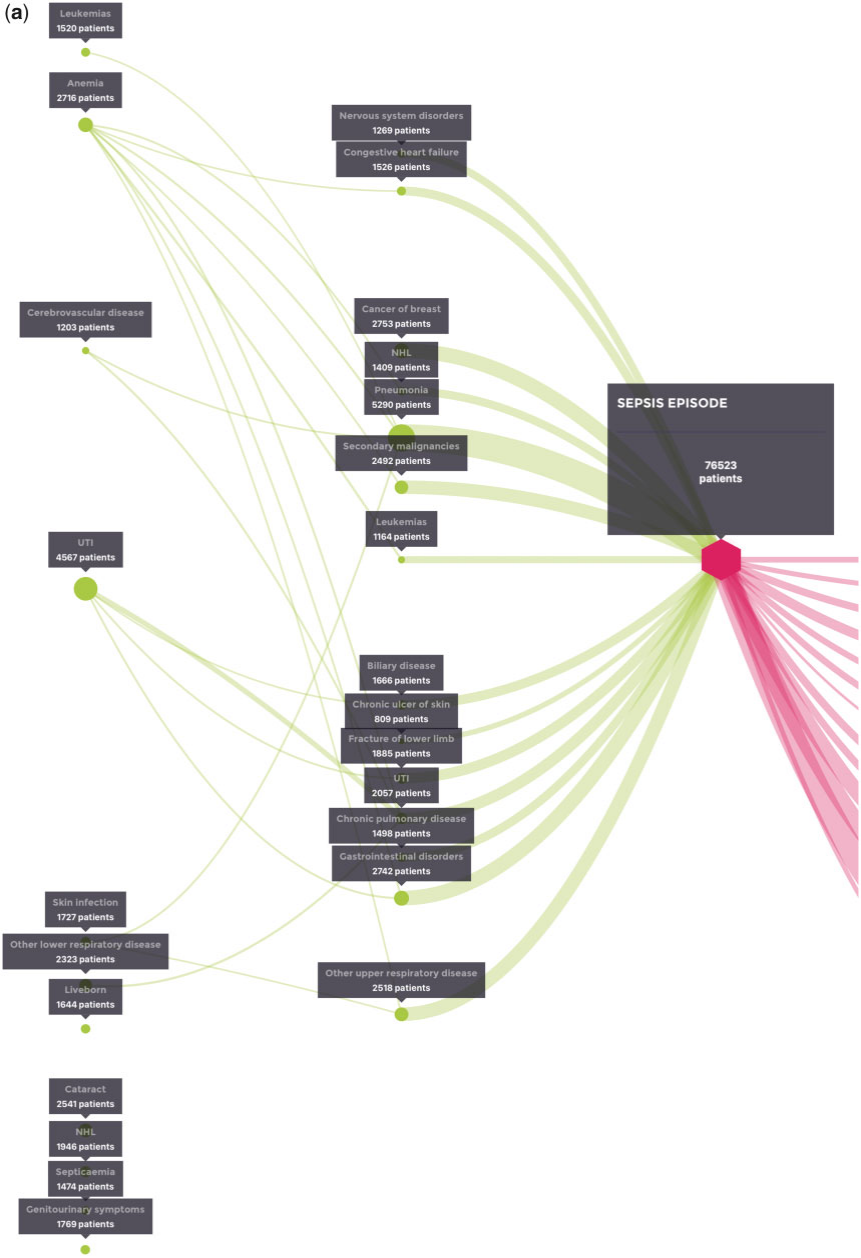
(A) Coded events in the 2 years before the index hospitalization for sepsis (with patient numbers). (B) Coded events in the 1 year after the index hospitalization for sepsis (with patient numbers).

**Figure 3.**
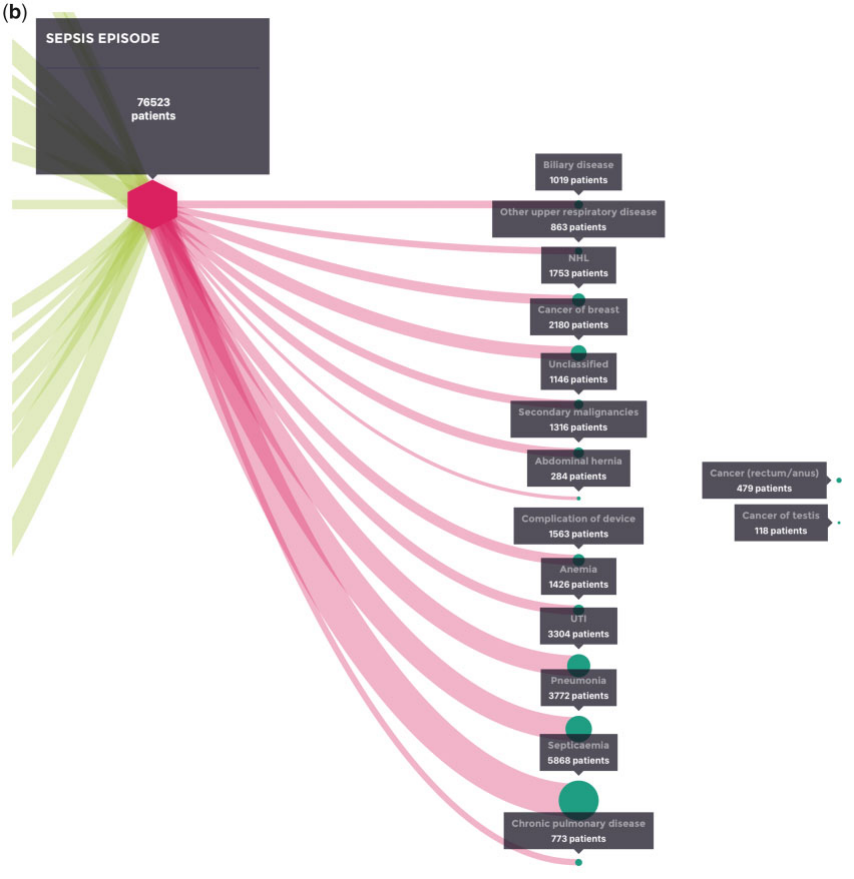
continued

The general prevalence and burden of comorbidities including cancer, respiratory/pulmonary diseases, cerebrovascular disease, cataract, and cardiovascular events were as expected, matching the expectation that sepsis usually occurs in vulnerable populations. Other diagnoses of interest that occurred with high enough frequency prior to the index sepsis event to appear in the model included nervous system disorders (1 269 patients), biliary disease (1 666 patients), skin infection (1 727 patients), fracture of the lower limb (1 885 patients), and genitourinary symptoms (1 769 patients).

Recurrent admission for UTI within 2 years also preceded sepsis hospitalization. A total of 5 854 patients had at least one admission for UTI before their index sepsis episode, and 770 patients had at least two admissions. To a lesser extent, hospitalization for either UTI or anemia, followed by hospitalization within 2 years for another cause (eg, biliary disease, fracture of the lower limb, or gastrointestinal disorder) also preceded the index hospitalization for sepsis with high frequency.

Table 3 shows the allocation of the top 10 SOS codes^12^ to the coded event categories. Nine of the 10 SOS codes are represented in the final model, with the exception of aspiration pneumonitis. Pneumonia, the most prevalent event prior to the sepsis index event, represents three of the top four codes, and UTI, the second most prevalent coded event, captured the third ranked SOS code.

**Table 3.** Allocation of the top 10 SOS codes to the coded event categories

Coded event class categories	Suspicion of sepsis codes
Pneumonia	J18.1 Lobar pneumonia, unspecified J18.9 Pneumonia, unspecified J18.0 Bronchopneumonia, unspecified
Septicemia	A41.9 Sepsis, unspecified
Urinary tract infections (UTI)	N39.0 Urinary tract infection, site not specified
Aspiration pneumonitis	J69.0 Pneumonitis due to food and vomit
Chronic obstructive pulmonary disease and bronchiectasis (“chronic pulmonary disease”)	J44.0 Chronic obstructive pulmonary disease with acute lower respiratory infection
Other lower respiratory disease	J22.X Unspecified acute lower respiratory infection
Skin and subcutaneous tissue infections (“skin infection”)	L03.1 Cellulitis of other parts of limb
Other gastrointestinal disorders (“gastrointestinal disorders”)	K63.1 Perforation of intestine (non-traumatic)

Coded events used in the present study have been mapped to the top 10 SOS codes. These SOS codes provide an indication of patients at high risk of sepsis who should undergo proactive screening and are a key target for improving the detection and treatment of sepsis.^12^ Shaded boxes indicate the coded event classes represented in the final sepsis discovery model.

It should be noted that sepsis (ICD-10 A40/A41 and derivatives), the coded event “septicemia” and the suspicion of sepsis (SOS) codes each provided a slightly differing, but overlapping, data capture for sepsis. This illustrates the ongoing challenges with achieving universal agreement on the definitions and coding for sepsis. A figure depicting the overlap of the codes within each of these diagnostic groupings for sepsis is provided in the Supplementary Materials.

Recurrence of septicemia, recurrence of pneumonia or recurrence of a UTI were the most common coded diagnoses for rehospitalization in the year after the index hospitalization event for sepsis (Figure 3B). There was no clear linkage (pathway) between specific health events after the sepsis index episode, indicating that at a population level each of the listed events were equally as likely to occur as the other.

## DISCUSSION

PM offers the opportunity to generate clinical and disease insights from large healthcare datasets. In England, the HES database is a large, national secondary care activity dataset. This proof of concept analysis confirms that a “bow-tie” pathway discovery analysis can be successfully applied to the HES database.^5^

During this feasibility analysis, the data structuration algorithm and visualization protocols were applied to more than 76 000 individual patient pathways related to an index sepsis hospitalization episode in 2016 (ICD-10 A40 or A41, and their derivatives). The key objectives of any assessment and improvements to the sepsis clinical management pathway are to better understand ways in which to identify patients at risk of either a primary sepsis event or a relapse (recurrence/reinfection) and to undertake early preventative intervention.^15^^,^^19^^,^^20^ Part of the challenge of studying the sepsis disease pathway is the diversity of conditions underlying its development, as well as the varying operational definitions of sepsis currently in use. This can often result in inconsistent coding of sepsis, with the potential for either underestimation or overestimation of sepsis events. Physicians rarely document (code) sepsis on admission, focusing instead on identifying the underlying infection, which in some cases may result in an under-reporting. There was a change in HES coding practices for sepsis in 2017 to reclassify patients with urosepsis and record this as sepsis secondary to a UTI, which resulted in a steep rise in numbers recorded. It is now believed that this likely resulted in an over-reporting of sepsis^19^ and is one of the main reasons why the 2016 study period was selected for this analysis.

The large size of the HES database is a strong advantage for its use in PM, particularly as all hospital admissions are detailed. However, the complexity of HES data meant that multiple steps were required to prepare and simplify the dataset to ensure the outputs can be interpreted (eg, aggregating ICD-10 codes into 220 coded event classes). As this was an initial feasibility analysis, a basic event coding protocol was undertaken and data management focused on technical aspects related to event labeling. There was less focus on defining detailed clinical and sepsis-data management rules or collecting demographic variables. This meant that there were limitations to the sepsis patient pathway insights that could be derived from the data. The limitation of the HES database to capture relevant hospital procedures should also be noted.

Preparing HES data for analysis required the creation of a large number of unique time-ordered diagnostic datasets corresponding to individual HES IDs. The time stamp selected for this was the principal diagnosis (main medical reason for admission) within a single hospital stay. Identify key diagnostic “milestones” that might expose sequential diagnostic linkages on the pathway toward sepsis and diagnostic events (outcomes) after sepsis. This evaluation is not dissimilar to the approach taken by Jensen et al^21^ to identify disease (diagnostic) trajectories from a population-wide registry data set in Denmark. A feasibility assessment of applying PM algorithms to evaluating the disease trajectories outlined by Jensen et al has more recently been undertaken by Kusuma et al,^6^ and our application of a PM discovery algorithm to identifying diagnostic trajectories for sepsis using an existing electronic health dataset provides further identification of the potential of this approach. The timeframe selected was 2 years prior and 1 year following a sepsis diagnosis. One limitation of this particular timeframe and the hospital stay time stamp is that it is unlikely to capture the rapid escalation in disease progression toward an acute sepsis event (eg, following surgery or chemotherapy). Consequently, a shorter time window (weeks or months), and potentially additional datasets, might be needed to find these specific “acute” candidate trigger events in the future.

It is also a limitation of the current discovery model for sepsis that time between events is not captured. From a medical point of view, it is extremely important to understand the relative timing of events, and which particular events and diagnoses place the patient at increased risk of sepsis. A similar process model structure—the Time Grid Process Model—has recently been introduced by De Oliveira et al,^22^ where the time patterns are analyzed during model optimization, and characteristic time distributions are represented in the final discovered model. It may be beneficial to also apply this model to the sepsis pathway in order to address specific questions of interest.

In order to achieve an effective trade-off between data complexity and visualization, and to balance the potential for either underfitting or overfitting the model, the number of events nodes included in the sepsis discovery models were constrained. These constraints could be released for future analysis, but at the potential risk of decreasing readability and interpretability. If, in the future, a specific focus on atypical pathways is undertaken it may be interesting to increase the number of event nodes, but we believe that these were an appropriate trade-off within the scope of the current work. It should be noted that the lack of presentation of a specific coded event in the model does not mean that this was not an event in a number of patient pathways. However, it did not occur with enough frequency (and/or consistency) to be represented in the final sepsis discovery model.

Despite such limitations, the final sepsis discovery model confirmed the clinical validity of the “bow-tie” pathway discovery analysis for sepsis. The model demonstrated a prevalence and burden of comorbidities that matched expectations, including patients known to be in compromised health, such as immunosuppression from cancer treatments. In addition, the most common pre-sepsis diagnostic events represented in the final model related to pneumonia and UTI, and covered the top four SOS codes, previously communicated by Inada-Kim et al.^15^ However, it should be noted that the pneumonia and UTI coded events also included 54 and 35 different ICD-10 codes, respectively, which may also explain why they each represent a significant number of events in the sepsis patient pathway. In future models, there would be the option to separate these out into more targeted events. Gastrointestinal disorders also featured highly as a pre-sepsis event, affecting 2742 patients, making it the third highest event after pneumonia and cancer of the breast. Again, this is in line with the expected risk factors for sepsis—the recent Global Burden of Disease Study has confirmed that diarrheal diseases remain the leading cause of sepsis worldwide, followed by lower respiratory infections, which also registered strongly in the model.^13^

Given the continuing lack of information as to which key factors and indicators can be used to best predict the risk of sepsis and how to prioritize patients for a higher level of care, some of the diagnostic patterns discovered in the analysis, such as recurrent hospitalization for UTI, may be clinically relevant predictors of sepsis worthy of further investigation. The model may also help to direct attention toward specific patient cohorts who could be considered at risk of sepsis and worthy of specific monitoring and attention. There are a number of obvious populations in which physicians currently remain alert for infections and sepsis, such as immunocompromized patients, but there may be other populations which are more frequently overlooked and could be better supported and/or targeted. This could include patients entering hospital with a community-acquired infection, or those at high risk of a hospital-acquired infection whilst undergoing clinical care for another medical condition. For example, patients with a history of either a UTI or anemia who are then hospitalized due to a physical trauma, such as a broken limb, who then go on to experience sepsis. Future versions of the model could examine such patient populations in more detail.

Finally, it is important to note that a further limitation for future analysis using HES database is that only secondary care (hospital) episodes are included within the database. Consequently, this analysis reflects only part of the health and care pathway for people developing and recovering from sepsis and does not represent events that may be important predictors or outcomes of sepsis outside of a hospital stay. Linkage of healthcare data for primary, secondary and social care healthcare datasets is becoming increasingly possible in the United Kingdom, for example the SAIL databank in Wales^23^ and the North West London (NWL) Whole Systems Integrated Care dataset (WSIC).^24^ It will be of great interest to evaluate the potential application of using these combined datasets in the future. This opens up multiple opportunities for process pathway discovery using healthcare datasets in England and the United Kingdom that could be explored.

## CONCLUSION

This study demonstrates that a PM pathway discovery analysis of the England HES database can be undertaken using a metaheuristic optimization algorithm.^5^ This can provide healthcare professionals, health system managers, and patients with optimal pathway discovery tools to examine key questions that could help to deliver improvements in clinical pathways of care.

A feasibility assessment was undertaken for the clinical pathway for sepsis, a life-threatening event that is often preceded by a complex and heterogeneous sequence of patient risk factors, healthcare events, and prior diagnoses. It demonstrated that the medical history of patients in the HES database with a diagnosis of sepsis could be effectively processed, evaluated, and visualized in order to help enable key diagnostic patterns and events to be discovered. It is hoped that this approach will lead to specific insights that, with further study, can be used to enable earlier intervention on the disease trajectory toward sepsis, thereby improving outcomes. The algorithm, data structuration protocol, and analytic techniques can now be more widely applied to the HES database to undertake targeted pathway discovery analysis across other healthcare conditions, with particular focus on evaluations with a central event or diagnosis, for example a stroke or a cardiovascular event.

## FUNDING

This work was supported by the Imperial College Health Partners.

## AUTHOR CONTRIBUTIONS

The study was initiated by AO, SB, LL, and MP. MIK, JW, and KA were members of the Steering Committee which oversaw the implementation of the study. Data extraction and analyses were performed by HDO and MP. All authors made substantial contributions to conception and design, acquisition of data, or analysis and interpretation of data: LL, AO, RS, S.SNOW, and S.SEKELJ closely worked with MIK and KA to design the algorithm for case extraction and to identify relevant medical outcomes of the study; HDO and MP designed the statistical analysis plan in accordance. All 11 authors contributed to the interpretation of the study and to the decision to publish the results. Preparation of the study manuscript was coordinated by RS and HDO and the writing was shared and revised among authors based on their field. All authors were involved in drafting the article or revising it critically for important intellectual content and gave final approval of the version to be published. All authors agree to be accountable for all aspects of the work in ensuring that questions related to the accuracy or integrity of any part of the work are appropriately investigated and resolved.

## SUPPLEMENTARY MATERIAL

Supplementary material is available at *Journal of the American Medical Informatics Association* online.

## CONFLICT OF INTEREST STATEMENT

None declared.

## Supplementary Material

ooaa039_Supplementary_Data
