# Supplementary material for: “Bow-tie” optimal pathway discovery analysis of sepsis hospital admissions using the Hospital Episode Statistics database in England
Source: JAMIA Open. 2020 Sep 20;3(3):439–48. doi: 10.1093/jamiaopen/ooaa039 (PMC7660952; doi:10.1093/jamiaopen/ooaa039)
Supplement: ooaa039_Supplementary_Data [file ooaa039_supplementary_data.pdf]

**Supplementary materials 1:**

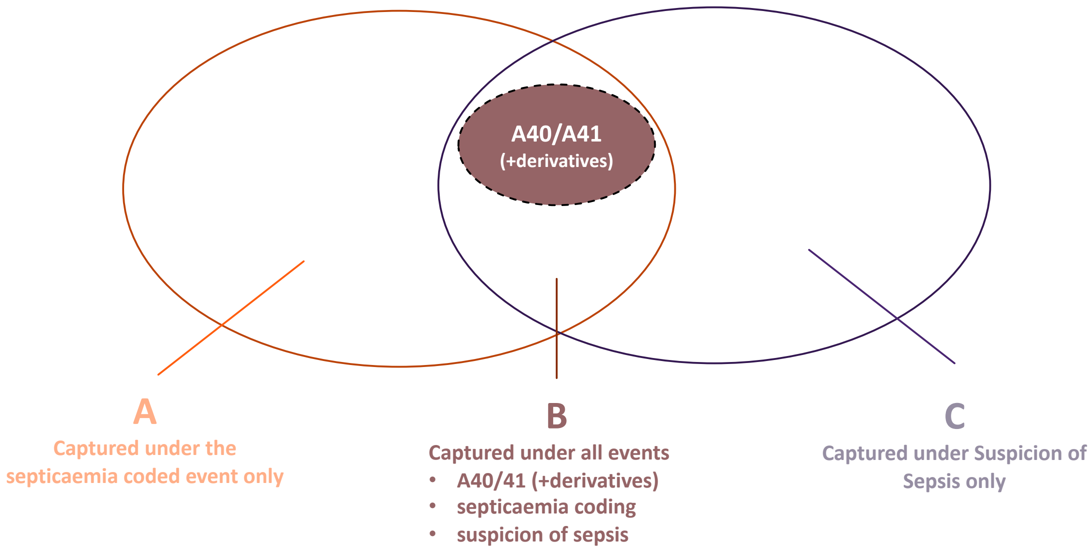


**Venn diagram representing ICD codes captured by the different sepsis naming**

See accompanying table for full details of the ICD-10 codes used under each coding category.

| **A. Captured under the septicaemia coded event ONLY** | | **B. Captured under all events – 1) A40/41 (+derivatives) and septicaemia coding (UNBOLDED) and 2) A40/41 (+derivatives), septicaemia coding and suspicion of sepsis (BOLD ONLY)** | | **C. Captured under Suspicion of Sepsis ONLY** | |
|---|---|---|---|---|---|
| 'A021' | Salmonella sepsis | 'A400' | Sepsis due to streptococcus, group A | J18.1 | Lobar pneumonia, unspecified |
| 'A207' | Septicemic plague | 'A401' | Sepsis due to streptococcus, group B | J18.9 | Pneumonia, unspecified |
| 'A227' | Anthrax sepsis | 'A403' | Sepsis due to Streptococcus pneumoniae | J18.0 | Bronchopneumonia, unspecified |
| 'A267' | Erysipelothrix sepsis | 'A408' | Other streptococcal sepsis | N39.0 | Urinary tract infection, site not specified |
| 'A327' | Listerial sepsis | 'A409' | Streptococcal sepsis, unspecified | J69.0 | Pneumonitis due to food and vomit |
| 'A392' | Acute meningococcemia | 'A4101' | Sepsis due to Methicillin susceptible Staphylococcus aureus | J44.0 | Chronic obstructive pulmonary disease with acute lower respiratory infection |
| 'A393' | Chronic meningococcemia | 'A4102' | Sepsis due to Methicillin resistant Staphylococcus aureus | J22.X | Unspecified acute lower respiratory infection |
| 'A394' | Meningococcemia, unspecified | 'A411' | Sepsis due to other specified staphylococcus | L03.1 | Cellulitis of other parts of limb |
| 'A021' | Salmonella sepsis | 'A412' | Sepsis due to unspecified staphylococcus | K63.1 | Perforation of intestine (non-traumatic) |
| 'A207' | Septicemic plague | 'A413' | Sepsis due to Hemophilus influenzae | | |
| 'A227' | Anthrax sepsis | 'A414' | Sepsis due to anaerobes | | |
| 'A267' | Erysipelothrix sepsis | 'A4150' | Gram-negative sepsis, unspecified | | |
| 'A327' | Listerial sepsis | 'A4151' | Sepsis due to Escherichia coli [E. coli] | | |
| 'A392' | Acute meningococcemia | 'A4152' | Sepsis due to Pseudomonas | | |
| 'A393' | Chronic meningococcemia | 'A4153' | Sepsis due to Serratia | | |
| 'A394' | Meningococcemia, unspecified | 'A4159' | Other Gram-negative sepsis | | |
| 'A021' | Salmonella sepsis | 'A4181' | Sepsis due to Enterococcus | | |
| 'A207' | Septicemic plague | 'A4189' | Other specified sepsis | | |
| 'A227' | Anthrax sepsis | **'A419'** | **Sepsis, unspecified organism** | | |
| 'A267' | Erysipelothrix sepsis | | | | |
| 'A327' | Listerial sepsis | | | | |
| 'A427' | Actinomycotic sepsis | | | | |
| 'A5486' | Gonococcal sepsis | | | | |
| 'B007' | Disseminated herpesviral disease | | | | |
| 'B377' | Candidal sepsis | | | | |
| 'I76' | Septic arterial embolism | | | | |

| | | | | | |
|---|---|---|---|---|---|
| 'P360' | Sepsis of newborn due to streptococcus, group B | | | | |
| 'P3610' | Sepsis of newborn due to unspecified streptococci | | | | |
| 'P3619' | Sepsis of newborn due to other streptococci | | | | |
| 'P362' | Sepsis of newborn due to Staphylococcus aureus | | | | |
| 'P3630' | Sepsis of newborn due to unspecified staphylococci | | | | |
| 'P3639' | Sepsis of newborn due to other staphylococci | | | | |
| 'P364' | Sepsis of newborn due to Escherichia coli | | | | |
| 'P365' | Sepsis of newborn due to anaerobes | | | | |
| 'P368' | Other bacterial sepsis of newborn | | | | |
| 'P369' | Bacterial sepsis of newborn, unspecified | | | | |
| 'R6520' | Severe sepsis without septic shock | | | | |
